# Assessment of clinical relevance of antigen improves diagnostic accuracy of hypersensitivity pneumonitis

**DOI:** 10.1186/s12890-024-02849-6

**Published:** 2024-02-14

**Authors:** Yuki Iijima, Masaru Ejima, Takashi Yamana, Shiro Sonoda, Sho Shibata, Tsuyoshi Shirai, Tsukasa Okamoto, Haruhiko Furusawa, Tomoya Tateishi, Takuya Adachi, Mio Mori, Susumu Kirimura, Tatsuhiko Anzai, Kunihiko Takahashi, Yasunari Miyazaki

**Affiliations:** 1 Department of Respiratory Medicine, Tokyo Medical and Dental University, 1-5-45, Yushima, Bunkyo-Ku, Tokyo 113-8519 Japan; 2 Department of Respiratory Medicine, Japanese Red Cross Musashino Hospital, 1-26-1, Minamimachi, Musasshino-City, Tokyo 180-8610 Japan; 3 Department of Pulmonary Immunotherapeutics, Tokyo Medical and Dental University, 1-5-45, Yushima, Bunkyo-Ku, Tokyo 113-8519 Japan; 4 Department of Diagnostic Radiology, Tokyo Medical and Dental University, 1-5-45, Yushima, Bunkyo-Ku, Tokyo 113-8519 Japan; 5 Department of Pathology, Tokyo Medical and Dental University, 1-5-45, Yushima, Bunkyo-Ku, Tokyo 113-8519 Japan; 6 Department of Biostatistics, Tokyo Medical and Dental University, 1-5-45, Yushima, Bunkyo-Ku, Tokyo 113-8519 Japan

**Keywords:** Interstitial lung disease, Hypersensitivity pneumonitis, Exposure assessment form, Antigen avoidance, Multidisciplinary discussion

## Abstract

**Background:**

Exposure assessment is integral to the diagnosis of hypersensitivity pneumonitis (HP). Although the clinical relevance of exposed antigens is essential for the assessment, many of the previous guidelines or reports have only evaluated simple exposure histories or immunological tests. To overcome this problem, the Exposure Assessment Form (EAF) was developed as an assessment tool for classifying the exposure grade from G0 to G4. The EAF was modified from the description in the Japanese clinical practice guide 2022 for HP published by the Japanese Respiratory Society.

**Methods:**

One hundred and seventy-two consecutive patients with interstitial lung disease who underwent multidisciplinary discussion (MDD) at our hospital were retrospectively examined. We assessed whether the use of the EAF improved the diagnostic performance of the international guideline of HP. We also evaluated whether the exposure grade affected the prognosis of HP.

**Results:**

Even when a HP diagnosis was made with a confidence of 70% or higher according to the international guideline, less than half of these cases resulted in a final diagnosis of HP when the exposure grades were lower than G3. When the result of the EAF was integrated into the exposure definition of the international guideline, the specificity of the diagnostic performance improved, while sensitivity was maintained. Furthermore, HP patients with an exposure grade of G3 or higher showed a tendency to take a longer time to initiate medication.

**Conclusions:**

This is the first study to evaluate the clinical relevance of possible antigens using the EAF. Assessing the exposure grade prevents overdiagnosis and improves the diagnostic performance of the international guideline.

**Supplementary Information:**

The online version contains supplementary material available at 10.1186/s12890-024-02849-6.

## Introduction

Hypersensitivity pneumonitis (HP) is a type of interstitial lung disease (ILD) caused by type III and type IV allergic alveolitis and progressive fibrosis. Because pathogenesis is triggered by sensitization to the inciting antigen (IA), avoidance of culprit exposure is a mainstay of treatment [1]. Since HP shares common features with other acute and chronic ILDs, such as idiopathic pulmonary fibrosis or other idiopathic interstitial pneumonias [2], diagnosis is difficult and requires a comprehensive evaluation based on clinical background, imaging, and pathological findings. In particular, the identification of the IA is central to the clinical domain of these diagnostic processes [3–5]. However, even with a thorough history, some potential exposures may be overlooked. Indeed, several reports indicate that nearly half of the cases of HP are attributable to unknown exposures [6, 7]. On the other hand, when several antigens are suspected simultaneously, it is difficult to specify which one is truly causative.

Recently, two international guidelines for the diagnosis of HP have been published [3–5], in which diagnosis is based on a combination of exposure assessment, bronchoalveolar lavage fluid (BALF) lymphocytosis, imaging, and histopathological findings. In the American Thoracic Society/Japanese Respiratory Society/Asociación Latinoamericana del Tórax guideline (ATS/JRS/ALAT-GL), exposure was defined as a history of exposure or a positive serum IgG test, which is a vague definition and is dependent on a subjective judgement by the clinician [3]. The following year, the CHEST guideline (CHEST-GL) additionally required sufficient evidence of an association between exposure and lung disease for the criteria of "identified antigen". However, it did not explicitly describe how to prove the "sufficient evidence" [4].

**Fig.1:**
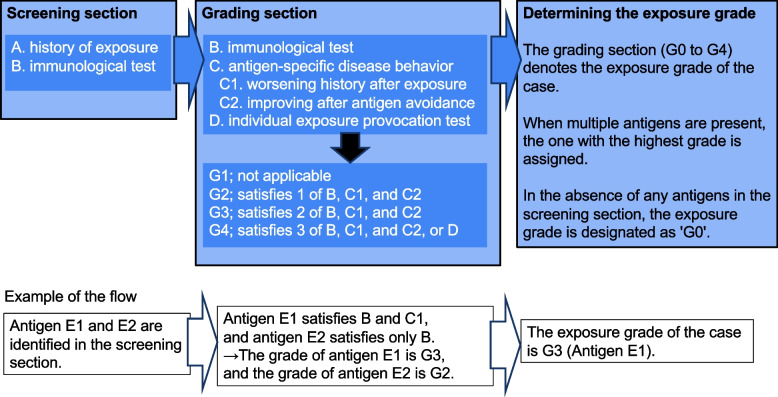
Flow of determining exposure grade using the EAF. In the screening section, antigen screening is performed based on simple exposure histories (item A) and immunological tests (item B). In the grading section, immunological tests (item B), association between exposure and disease behavior (item C), and individual exposure provocation tests (item D) are evaluated to classify the clinical relevance of the antigen into grades G0 to G4. The antigen with the highest grade is considered the most relevant antigen, and this grade is recorded as the exposure grade for the patient. When no antigen is identified in the screening section, the exposure grade is reported as G0

IAs known to cause HP, such as avian or fungal antigens, are widely present in our environment. Therefore, a simple exposure history is insufficient for estimating the clinical relevance of an IA, and its association with disease behavior should also be assessed. From this perspective, the Exposure Assessment Form (EAF) was developed to classify clinical relevance of an IA into 4 grades from G1 to G4, which lead to the assessment of the exposure grade of the case into 5 grades from G0 to G4 (Fig. 1, Table S1). This classification was modified from the description in the Japanese clinical practice guide 2022 for hypersensitivity pneumonitis published by the Japanese Respiratory Society [8]. In this study, we examined how the use of the EAF impacts the accurate diagnosis of HP.

## Methods

### Outline of the study

First, we retrospectively reviewed ILD cases examined at our institute. For each case, the exposure grade was determined based on the result of the EAF. Two types of diagnoses were also made including a diagnosis based on the ATS/JRS/ALAT-GL (GL diagnosis) and a final diagnosis based on multidisciplinary discussion (MDD). Then, we investigated whether the integration of the exposure grade into the ATS/JRS/ALAT-GL could enhance its accuracy in predicting the final diagnosis. Finally, the impact of the exposure grade on the clinical outcome of HP was evaluated.

### Exposure grade

The EAF is composed of a screening section (Items A and B) and a grading section (Items B, C, and D; Item B is included in both sections) (Fig. 1, Table S1). In the screening section, potential antigens are screened from the medical history in Item A and the immunological evidence in Item B [9, 10]. In the grading section, the screened antigens are graded by their clinical relevance. Item B has a role not only in screening but also in grading. The immunologic findings included results from commercially available tests in Japan for anti-Trichosporon asahii antibodies (enzyme-linked immunosorbent assay; ELISA) and serum-specific IgG antibodies against budgerigar and pigeon (ImmunoCAP®, ThermoFisher) [10]. Additionally, results of an in-house ELISA for pigeon droppings extract were also included [9]. In Item C, the association between exposure and disease behavior is evaluated, which is further divided into 2 subitems, C1 and C2. Subitem C1 is considered to have a positive result when there is a history of worsening after new or increased exposure. For example, the following situations fall into this category: apparent radiological worsening or elevation of ILD-specific biomarkers such as Krebs von den Lungen 6 or surfactant protein-D were observed after an episode of exposure, serial changes in these biomarkers or lung function showed seasonal variation which is specific for the antigen [11, 12], or the environmental provocation test was positive. Subitem C2 is considered to have a positive result when antigen avoidance improves lung disease. For example, an apparent decrease in ILD-specific biomarkers or an apparent improvement in lung function after abatement of the antigen falls into this category [13]. Item D is an individual exposure provocation test, which is distinguished from the environmental provocation test in subitem C1 [14]. Then, the clinical relevance of each antigen is classified into 4 grades based on a combination of these Items: G1 (not evident), G2 (weak suspicion), G3 (strong suspicion), and G4 (confirmed). For each patient, the antigen with the highest grade was considered the most relevant, and the grade for this antigen was reported as the “exposure grade” of the patient. When no antigen was identified in the screening section, the exposure grade was reported as G0 (Fig. 1, Table S1).

### Study population and data collection

The study population included 172 consecutive patients with ILD diagnosed by MDD at Tokyo Medical and Dental University Hospital, Tokyo, from September 1st, 2020, to June 30th, 2023. Clinical data, including age, sex, spirometry, BALF cell count, and EAF result, were collected from medical records. In addition, radiological classification, pathological classification, and GL diagnosis were made according to the ATS/JRS/ALAT-GL algorithm. The observation period was calculated from the date of the MDD until the last visit (date of censoring or death). This study on humans was conducted according to guidelines of the Declaration of Helsinki and approved by the Ethics Committee of Tokyo Medical and Dental University (approval number M2019-206). Informed consent was waived by Ethics Committee of Tokyo Medical and Dental University because of the retrospective nature of the study.

### Diagnosis

GL diagnoses and final diagnoses were made for all cases. The MDD team was composed of at least 5 respiratory, radiology, and pathology experts, who provided a diagnosis and confidence level. As this was a retrospective study, two different ontological frameworks were used between the GL diagnosis and the final diagnosis. According to the ATS/JRS/ALAT-GL^3^, the confidence of GL diagnosis was based on a 5-level ontological framework, in which 90% or higher was considered “definite diagnosis”, 80–89% was “high confidence”, 70–79% was “moderate confidence”, 50–69% was “low confidence”, and less than 50% was “not excluded (NE)”. In contrast, the confidence of the final diagnosis was based on a 4-level ontological framework presented by Ryerson et al. [15], in which 90% or higher was considered “definite diagnosis”, 70–89% was “high confidence”, 50–69% was “low confidence”, and less than 50% was “other diagnosis”. Consequently, a high confidence in the final diagnosis is equivalent to moderate and high confidence in the GL diagnosis.

### Statistical analysis

Continuous variables are expressed as the means ± standard deviations (SD). The chi-squared test for trend in proportions was used to compare the frequency of confidence change of diagnosis between exposure grades. The log-rank test was used to compare the survival curves of patients stratified by exposure grade. All statistical analyses were carried out using Stata 17.0 (Stata Corp., College Station, TX, USA), and *p* values of < 0.05 were considered significant.

## Results

### Patient characteristics

The mean age of the patients was 64.6 years, and approximately 60% were male. Surprisingly, 167 of 172 patients (97.1%) met the "with exposure" criteria in the ATS/JRS/ALAT-GL, which corresponded to a G1 or higher exposure grade according to the EAF. Between GL diagnosis and final diagnosis, there was a decrease in the number of patients with a diagnostic confidence of 50–89%, while an increase was observed in the number of patients with a diagnostic confidence of 49% or below. On the other hand, the number of patients with a diagnostic confidence of 90% or higher remained almost unchanged (Table 1).

**Table 1:** Patient characteristics (*n* = 172)

Age	64.6 ± 12.4	Diagnostic confidence of HP (ATS/JRS/ALAT-GL)	
Male	103 (59.9%)	90%- (Definite)	28 (16.3%)
%FVC (%)	82.3 ± 16.0	70–89% (High + Moderate)	80 (46.5%)
Fibrotic/nonfibrotic	147/25	50–69% (Low)	46 (26.7%)
With exposure (ATS/JRS/ALAT-GL)	167 (97.1%)	-49% (NE)	18 (10.5%)
Exposure grade (EAF)		Diagnostic confidence of HP (Final diagnosis by MDD)	
G0/G1/G2/G3/G4	5/63/66/33/5	90%- (Definite)	33 (19.2%)
BALF cell count		70–89% (High)	33 (19.2%)
Lym≧30%, < 30%, NA	36/120/16	50–69% (Low)	13 (7.6%)
HRCT classification (ATS/JRS/ALAT-GL)		-49% (others^a^)	93 (54.1%)
Typical/Compatible/Indeterminate	31/113/28		
Pathology classification (ATS/JRS/ALAT-GL)			
Typical/Probable/Indeterminate/No histopathology	13/81/73/5		

*ATS/JRS/ALAT-GL* American Thoracic Society/Japanese Respiratory Society/Asociación Latinoamericana del Tórax guideline, *BALF* bronchoalveolar lavage fluid, *NA* not assessed, *HRCT* high resolution computed tomography, *NE* not excluded, *MDD* multidisciplinary discussion

^a^Others include idiopathic pulmonary fibrosis (*n* = 2), idiopathic nonspecific interstitial pneumonia (*n* = 26), collagen vascular disease related interstitial lung disease (*n* = 4), unclassifiable interstitial lung disease (*n* = 40), smoking-related interstitial lung disease (*n* = 8), Idiopathic pleuroparenchymal fibroelastosis (*n* = 4), lymphoproliferative disease (*n* = 2), sarcoidosis (*n* = 2), inflammatory change (*n* = 1), pulmonary artery infarction (*n* = 1), chronic eosinophilic pneumonia (*n* = 1), eosinophilic granulomatosis with polyangiitis (*n* = 1), and drug-induced interstitial lung disease (*n* = 1)

### Identified antigens

In the screening section of the EAF, a total of 334 antigens were screened by Items A (clinical history of exposure) and B (immunological evidence of exposure). The mean number of screened antigens was 1.9 per patient, while no antigen was identified in only 5 patients. The most common antigen was avian. In the grading section, Item B was the most common (30.5%), followed by subitem C2 (25.1%), subitem C1 (9.0%), and item D (0.3%). Consequently, approximately half of the antigens were graded as G1, followed by G2 (35.9%), G3 (12.6%), and G4 (1.5%) (Table S2).

### Impacts of the exposure grade on the final diagnosis

Although 108 patients were diagnosed with HP with a threshold of 70% confidence in the ATS/JRS/ALAT-GL (GL-HP), the final diagnosis was often different if G3 or higher antigens were absent. Namely, this group included 43 patients with an exposure grade of G2 and 32 patients with the grade of G1, of which respectively only 23 (53.5%) and 10 (31.3%) were finally diagnosed as HP higher than 70% confidence. Among the remaining 64 patients who did not meet the GL-HP criteria, the majority were finally diagnosed with etiologies other than HP, regardless of their exposure grade (Table 2). As the exposure grade decreased in each case, the confidence level in the final diagnosis showed a corresponding decrease from that of the GL diagnosis, indicating a correlation between exposure grade and diagnostic confidence (Table 3). These results suggest that the EAF is likely to prevent overdiagnosis by the ATS/JRS/ALAT-GL.

**Table 2:** The impacts of discrepancy between GL diagnosis and exposure grade on the final diagnosis

	Exposure grade	Final diagnosis of HP
GL-HP (*n* = 108)	G4 (*n* = 5)	5 (100%)
	G3 (*n* = 28)	26 (92.9%)
	G2 (*n* = 43)	23 (53.5%)
	G1 (*n* = 32)	10 (31.3%)
	G0 (*n* = 0)	0 (NA)
Other (*n* = 64)	G4 (*n* = 0)	0 (NA)
	G3 (*n* = 5)	1 (20.0%)
	G2 (*n* = 23)	1 (4.3%)
	G1 (*n* = 31)	0 (0%)
	G0 (*n* = 5)	0 (0%)

*GL* diagnosis, diagnosis based on the *ATS/JRS/ALAT-GL*, *EAF* exposure assessment form, *GL-HP* HP with a threshold of 70% confidence in the ATS/JRS/ALAT-GL, *NA* not applicable

**Table 3:** Decrease of confidence from GL diagnosis to final diagnosis

Exposure grade	Confidence increase or unchange	Confidence decrease	total
G4	4 (80.0%)	1 (20.0%)	5
G3	25 (75.8%)	8 (24.2%)	33
G2	28 (42.4%)	38 (57.6%)	66
G1	18 (28.6%)	45 (71.4%)	63
G0	5 (100%)	0 (0.0%)	5
Total	80	92	172

*p* < 0.01 (Chi-squared Test for Trend in Proportions)

### Integration of the EAF and ATS/JRS/ALAT-GL

Next, the EAF results were integrated into the ATS/JRS/ALAT-GL, resulting in the creation of three variations of the modified guideline: G2-mGL, G3-mGL, and G4-mGL. These variations required exposure grades of G2 or higher, G3 or higher, and G4 or higher, respectively, to meet the criteria of "with exposure" in the ATS/JRS/ALAT-GL. The diagnostic performance of these modified guideline was then compared to that of the ATS/JRS/ALAT-GL, using the final diagnosis as the gold standard.

At thresholds for meaningful diagnostic confidence of 50%, 70%, and 90%, the diagnostic sensitivity showed minimal changes in G2-mGL and G3-mGL, while it decreased in G4-mGL. The diagnostic specificity was highest in G4-mGL, followed by G3-mGL, G2-mGL, and ATS/JRS/ALAT-GL in that order, at any threshold. Consequently, G3-mGL appeared to be the best balance since it exhibited equivalent sensitivity and higher specificity compared to the ATS/JRS/ALAT-GL. The increase in specificity was especially apparent at thresholds of 50% and 70% confidence (Table 4).

**Table 4:** Diagnostic performance of modified GL

	Sensitivity	Specificity	PPV	NPV
Confidence ≧90% in ATS/JRS/ALAT-GL	0.58 [0.39–0.75]	0.94 [0.88–0.97]	0.68 [0.48–0.84]	0.90 [0.84–0.95]
Confidence ≧90% in G2-mGL	0.58 [0.39–0.75]	0.94 [0.89–0.98]	0.70 [0.50–0.86]	0.90 [0.84–0.95]
Confidence ≧90% in G3-mGL	0.52 [0.34–0.69]	0.95 [0.90–0.98]	0.71 [0.49–0.87]	0.89 [0.83–0.94]
Confidence ≧90% in G4-mGL	0.36 [0.20–0.55]	0.97 [0.93–0.99]	0.75 [0.48–0.93]	0.87 [0.80–0.92]
Confidence ≧70% in ATS/JRS/ALAT-GL	0.97 [0.89–1.00]	0.59 [0.49–0.68]	0.59 [0.49–0.69]	0.97 [0.89–1.00]
Confidence ≧70% in G2-mGL	0.97 [0.89–1.00]	0.67 [0.57–0.76]	0.65 [0.54–0.74]	0.97 [0.90–1.00]
Confidence ≧70% in G3-mGL	0.96 [0.87–0.99]	0.72 [0.63–0.81]	0.68 [0.57–0.77]	0.96 [0.89–0.99]
Confidence ≧70% in G4-mGL	0.91 [0.81–0.97]	0.72 [0.63–0.81]	0.67 [0.56–0.76]	0.93 [0.85–0.97]
Confidence ≧50% in ATS/JRS/ALAT-GL	1.00 [0.93–1.00]	0.19 [0.12–0.29]	0.51 [0.43–0.59]	1.00 [0.74–1.00]
Confidence ≧50% in G2-mGL	0.99 [0.93–1.00]	0.41 [0.31–0.52]	0.59 [0.50–0.67]	0.97 [0.87–1.00]
Confidence ≧50% in G3-mGL	0.95 [0.88–0.99]	0.58 [0.47–0.68]	0.66 [0.56–0.74]	0.93 [0.83–0.98]
Confidence ≧50% in G4-mGL	0.92 [0.84–0.97]	0.61 [0.51–0.71]	0.67 [0.57–0.76]	0.91 [0.80–0.96]

*ATS/JRS/ALAT-GL* American Thoracic Society/Japanese Respiratory Society/Asociación Latinoamericana del Tórax guideline, *PPV* positive predictive value, *NPV* negative predictive value

### Impacts of the exposure grade on disease progression

**Fig. 2:**
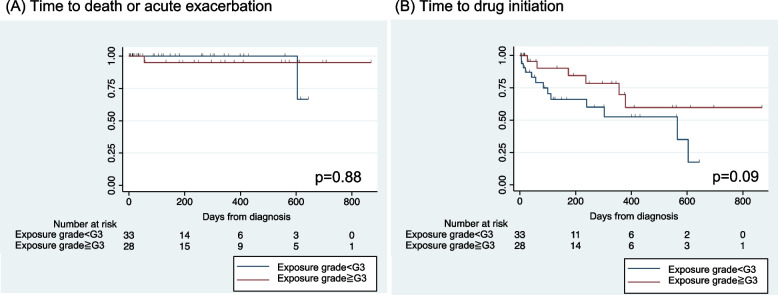
Effect of exposure grade on clinical outcome. Kaplan‒Meier survival curves of disease progression are shown. The time until death or acute exacerbation was not significantly different based on exposure grade (**A**). On the other hand, there was a tendency for the duration until the initiation of pharmacological treatment with steroids or antifibrotic agents to be longer in exposure grades G3 and higher (**B**)

The 66 patients with a final diagnosis of HP with confidence level of higher than 70% were segregated into two groups based on exposure grade using cut-off values of G2, G3, or G4. When the time to death or acute exacerbation was considered as the endpoint, there was no significant difference between the Kaplan‒Meier curves (Fig. 2, Fig. S1). However, when the time to initiate drug treatment, including steroids and antifibrotic agents, was the endpoint, the Kaplan‒Meier curve revealed a trend towards a prolonged time to reach the endpoint in patients with an exposure grade of G3 or higher (*p* = 0.09) (Fig. 2, Fig. S1).

## Discussion

In this study, we investigated the utility of the EAF as a clinical assessment for diagnosing HP. Identifying the IA is crucial for HP diagnosis, and the CHEST-GL requires sufficient evidence of an association between the antigen and lung disease. To define the association, the Japanese Respiratory Society proposed an assessment form in the Japanese clinical practice guide 2022 for hypersensitivity pneumonitis. The EAF is a modified version of this assessment form, aiming to provide a more practical approach. Even in cases with high confidence HP according to the ATS/JRS/ALAT-GL, the final diagnosis was often different if G3 or higher antigens were absent. When the criteria of “with exposure” according to the ATS/JRS/ALAT-GL was defined as an exposure grade of G3 or G4, the diagnostic specificity was improved. Furthermore, among the HP patients with more than 70% confidence, the time to drug initiation tended to be prolonged in cases where the exposure grade was G3 or higher.

Our results demonstrated that the EAF was useful, especially for preventing overdiagnosis according to the ATS/JRS/ALAT-GL. Previously, the exposure questionnaire was developed using a scoping systematic review and Delphi consensus method [16–18]. However, it is difficult to assign significance to antigens, which is one of the major barriers to antigen assessment [19]. As meeting criteria of “with exposure” increases the diagnostic likelihood of HP according to the ATS/JRS/ALAT-GL, simply using the questionnaire may potentially lead to overdiagnosis due to the presence of exposure unrelated to the disease. Indeed, in our study, as many as 97.1% of the cases were classified as "with exposure” in the definition of the ATS/JRS/ALAT-GL, despite the exposure history being collected using a questionnaire. Moreover, almost half of cases with a GL diagnosis with 70% or higher confidence were finally diagnosed differently in the absence of G3 or higher antigen. Accordingly, the EAF is meaningful for preventing an overestimation of the exposure assessment.

The integration of the EAF into the ATS/JRS/ALAT-GL did not improve diagnostic performance at thresholds for meaningful confidence level of 90%. However, the use of EAF improved the diagnostic performance at thresholds for meaningful confidence level of 50% or 70%. Specifically, the high diagnostic sensitivity of the ATS/ERS/ALAT-GL was maintained and the specificity was largely improved for G3-mGL. These results suggest two important points. First, the EAF is useful for diagnosis in cases where HP is suspected but a definitive diagnosis cannot be made. Second, among the exposure grades in the EAF, G3 may be the most balanced cut-off. In other words, for individual antigens, a significant association with the disease could be considered when meeting either two of the following findings: immunological evidence, worsening with antigen exposure, and improvement with antigen removal.

In our study, there was no association between exposure grade and mortality/adverse events in HP patients. However, G3 or higher exposure grades were associated with a tendency toward a longer time to intervention with drug therapy. This result is consistent with previous reports showing that antigen identification and avoidance are associated with improved HP prognosis [6, 7, 20, 21]. Therefore, the consistency indicates the validity of the classification of exposure grade using the EAF.

There are several limitations in our study. First, this is a single-center retrospective study. Second, Trichosporon asahii and avian antigens are commonly identified as causative antigens for HP in Japan, which make the item B focus on these antigens. However, due to regional variations in the prevalence of causative antigens, the specifications of item B should be changed accordingly. Third, the items included in the EAF are not defined in detail, which may result in discrepancies between assessments among observers. For example, subitem C1 does not define which biomarker and how much change is considered as worsening. For subitem C2, it is not clear whether complete avoidance or only a reduced amount of exposure is required for the evaluation. However, such detailed definitions may depend on various factors, including the type of antigen, the amount of exposure, or the severity of the disease. Since a larger study is needed to overcome this problem, and as this study was intended to introduce the concept of the EAF as a preliminary step, the judgement was left to the interpretation of the physicians without a detailed definition. Forth, the EAF grades were not associated with clinical outcomes such as death or acute deterioration in our study. Most previous reports had shown the correlation between antigen identification and prognosis with follow-up periods of at least 5 years or longer [6, 7, 22]. However, the maximum follow-up period in our study was approximately 2 years. Hence, an insufficient observation period may have resulted in a failure to show significant differences. Fifth, in comparing Kaplan–Meier curves for clinical outcomes, there were numerous instances of censoring, which also included cases that could no longer visit our hospital due to disease progression. Sixth, the greater the suspicion of HP by the attending physician, the more priority is given to follow-up with antigen avoidance prior to drug treatment, which may affect the timing of drug initiation as a competitive risk. Finally, antigen exposure history is also considered in MDD, which introduces an incorporation bias when using the final diagnosis as a gold standard.

In conclusion, this is the first study to evaluate the clinical relevance of IA. The EAF is a useful tool for assessing the clinical relevance of antigens and the exposure grade of individual cases, which may prevent HP overdiagnosis according to the ATS/JRS/ALAT-GL.

### Supplementary Information

**Additional file1:  Table S1.** Exposure Assessment Form (EAF).

**Additional file2:  Table S2.** Detail of antigens.

**Additional file3: Figure S1. **Effect of exposure grades including G2, G3, and G4 on clinical outcome.

## Data Availability

The datasets used and/or analyzed during the current study are available from the corresponding author on reasonable request.

## Abbreviations

HP: Hypersensitivity pneumonitis
ILD: Interstitial lung disease
IA: Inciting antigen
BALF: Bronchoalveolar lavage fluid
ATS/JRS/ALAT-GL: American Thoracic Society/Japanese Respiratory Society/Asociación Latinoamericana del Tórax guideline
CHEST-GL: CHEST guideline
EAF: Exposure Assessment Form
GL diagnosis: Diagnosis based on the ATS/JRS/ALAT-GL
MDD: Multidisciplinary discussion
ELISA: Enzyme-linked immunosorbent assay
NE: Not excluded
SD: Standard deviation
GL-HP: HP with a threshold of 70% confidence in the ATS/JRS/ALAT-GL
