# Supplementary material for: Assessment of clinical relevance of antigen improves diagnostic accuracy of hypersensitivity pneumonitis
Source: BMC Pulm Med. 2024 Feb 14;24:84. doi: 10.1186/s12890-024-02849-6 (PMC10865633; doi:10.1186/s12890-024-02849-6)
Supplement: Supplementary file 1 — Additional file1: Table S1. Exposure Assessment Form (EAF). [file 12890_2024_2849_MOESM1_ESM.docx]

Table S1. Exposure Assessment Form (EAF)

| **Exposure Assessment Form (EAF)** | | | | | |
| --- | --- | --- | --- | --- | --- |
| **Screening of antigen exposure** | | |  |  |  |
|  |  | **Grading of clinical significance (G1〜G4)** | | | |
| **Antigen** | **A Questionnaire and history taking** | **B Immunological findings** | **C Association with the clinical picture** | **D Reproducibility** | **Grade** |
| **Fungi/**  **molds** | ☐Location  （　　　　　　）  ☐Moldy odor  ☐Room with poor sunlight  ☐Room with dampness  ☐Leaks and flooding  ☐Wooden structure 20 years or more  ☐Others  (　　　　　　　　) | ☐Anti-*T. asahii*-Ab positive  ☐Others  (　　　　　　　　) | **C1 Suggests deterioration due to increased exposure**  ☐Worsening with contact  ☐Seasonal aggravation  ☐Worsening by return to the environment  **C2 Suggests improvement due to decreased exposure**  ☐ Antigen avoidance test positive  ☐ Stabilization after antigen removal | ☐Individual provocation test positive | ☐G1  ☐G2  ☐G3  ☐G4 |
| **Avian** | **Contact with birds**  ☐History of breeding  ☐Neighborhood breeding, sheds, nests  ☐Habits of feeding and contact  ☐Flying, feathers, droppings in the yard | Bird-specific IgG  ☐pigeon positive  ☐budgerigar positive    ☐Others  (　　　　　　　　) | **C1 Suggests deterioration due to increased exposure**  ☐Worsening with contact  ☐Seasonal aggravation  ☐Worsening by return to the environment  **C2 Suggests improvement due to decreased exposure**  ☐ Antigen avoidance test positive  ☐ Stabilization after antigen removal | ☐Individual provocation test positive | ☐G1  ☐G2  ☐G3  ☐G4 |
|  | **Feather products**  **(Ex; Down jacket or feather duvet, etc.）**  ☐Used by patient  ☐Used only by family members  ☐Storing |  |  |  |  |
|  | **poultry manure fertilizer**  ☐Use at home  ☐Use in the neighborhood |  |  |  |  |
|  | ☐**Others**  (　　　　　　　　) |  |  |  |  |
| **Humidifier** | ☐Ultrasonic type  ☐Heating type  ☐Other types |  | **C1 Suggests deterioration due to increased exposure**  ☐Worsening with contact  ☐Seasonal aggravation  ☐Worsening by return to the environment  **C2 Suggests improvement due to decreased exposure**  ☐ Antigen avoidance test positive  ☐ Stabilization after antigen removal | ☐Individual provocation test positive | ☐G1  ☐G2  ☐G3  ☐G4 |
| **Others**  **（           ）** | Details  (　　　　　　　　) | ☐Others  (　　　　　　　　) | **C1 Suggests deterioration due to increased exposure**  ☐Worsening with contact  ☐Seasonal aggravation  ☐Worsening by return to the environment  **C2 Suggests improvement due to decreased exposure**  ☐ Antigen avoidance test positive  ☐ Stabilization after antigen removal | ☐Individual provocation test positive | ☐G1  ☐G2  ☐G3  ☐G4 |
| **Grading of clinical significance**  ・G1 (not evident); None  ・G2 (weak suspicion);　　B or C1 or C2  ・G3 (strong suspicion);　  B+C1 or B+C2 or C1+C2  ・G4 (confirmed);　　　　  B+C1+C2 or D | | | | | |
