# Supplementary material for: Assessment of clinical relevance of antigen improves diagnostic accuracy of hypersensitivity pneumonitis
Source: BMC Pulm Med. 2024 Feb 14;24:84. doi: 10.1186/s12890-024-02849-6 (PMC10865633; doi:10.1186/s12890-024-02849-6)
Supplement: Supplementary file 2 — Additional file2: Table S2. Detail of antigens. [file 12890_2024_2849_MOESM2_ESM.docx]

Table S2. Detail of antigens

| Total number of antigens | 334 |
| --- | --- |
| Average number of antigens per case | 1.9 |
| Number of identified antigens per case (0/1/2/3/4) | 5/41/86/39/1 |
| Avian/Molds/Humidifier/Others | 158/115/57/4 |
| Number of positive antigens per grading item |  |
| B/C1/C2/D | 102 (30.5%)/30 (9.0%)/84 (25.1%)/1 (0.3%) |
| Number of antigens of per grade |  |
| G1/G2/G3/G4 | 167 (50.0%)/120 (35.9%)/42 (12.6%)/5 (1.5%) |
