# Supplementary material for: Assessment of clinical relevance of antigen improves diagnostic accuracy of hypersensitivity pneumonitis
Source: BMC Pulm Med. 2024 Feb 14;24:84. doi: 10.1186/s12890-024-02849-6 (PMC10865633; doi:10.1186/s12890-024-02849-6)
Supplement: Supplementary file 3 — Additional file3: Figure S1. Effect of exposure grades including G2, G3, and G4 on clinical outcome. [file 12890_2024_2849_MOESM3_ESM.docx]

**Figure S1. Effect of exposure grades including G2, G3, and G4 on clinical outcome**


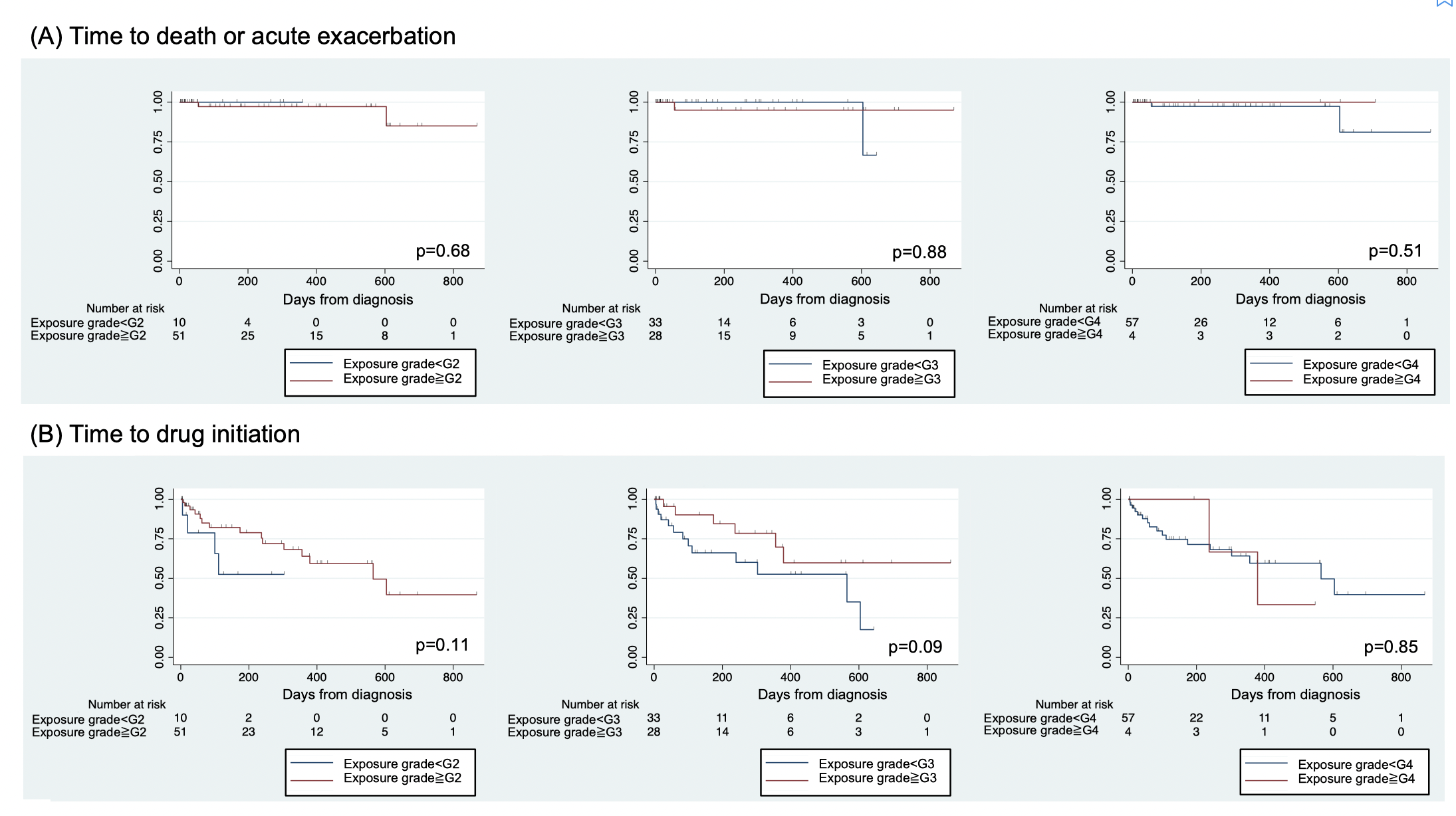


Kaplan‒Meier survival curves of disease progression are shown. In both (A) and (B), time to clinical outcome is compared with the cutoffs for exposure grade as G2, G3, and G4, from left to right. Panel (A) shows that the time until death or acute exacerbation did not differ significantly among the exposure grades. Conversely, panel (B) indicates a trend towards a longer duration before initiating pharmacological treatment with steroids or antifibrotic agents in patients with exposure grades G3 and above.
